# Prediction of skull fractures in blunt force head traumas using finite element head models

**DOI:** 10.1007/s10237-023-01768-5

**Published:** 2023-09-01

**Authors:** Natalia Lindgren, Mikkel J. Henningsen, Christina Jacobsen, Chiara Villa, Svein Kleiven, Xiaogai Li

**Affiliations:** 1 Division of Neuronic Engineering, KTH Royal Institute of Technology, Stockholm, Sweden; 2 Department of Forensic Medicine, University of Copenhagen, Copenhagen, Denmark

**Keywords:** Skull fracture, Finite element head model, Accident reconstruction, Injury prediction, Head injury mechanisms

## Abstract

Traumatic head injuries remain a leading cause of death and disability worldwide. Although skull fractures are one of the most common head injuries, the fundamental mechanics of cranial bone and its impact tolerance are still uncertain. In the present study, a strain-rate-dependent material model for cranial bone has been proposed and implemented in subject-specific Finite Element (FE) head models in order to predict skull fractures in five real-world fall accidents. The subject-specific head models were developed following an established image-registration-based personalization pipeline. Head impact boundary conditions were derived from accident reconstructions using personalized human body models. The simulated fracture lines were compared to those visible in post-mortem CT scans of each subject. In result, the FE models did predict the actual occurrence and extent of skull fractures in all cases. In at least four out of five cases, predicted fracture patterns were comparable to ones from CT scans and autopsy reports. The tensile material model, which was tuned to represent rate-dependent tensile data of cortical skull bone from literature, was able to capture observed linear fractures in blunt indentation loading of a skullcap specimen. The FE model showed to be sensitive to modeling parameters, in particular to the constitutive parameters of the cortical tables. Nevertheless, this study provides a currently lacking strain-rate dependent material model of cranial bone that has the capacity to accurately predict linear fracture patterns. For the first time, a procedure to reconstruct occurrences of skull fractures using computational engineering techniques, capturing the all-in-all fracture initiation, propagation and final pattern, is presented.

## Introduction

Despite considerable advancements in injury prevention in the past decades, traumatic head injuries remain a leading cause of death and disability worldwide (Faul and Coronado 2015; Schmitt et al. 2019). One of the most common head injury is skull fractures (Malczyk et al. 2014), which are likely accompanied by other severe injuries like subarachnoid-, subdural-, or epidural hemorrhages (Aminoff et al. 2015). Skull fractures are commonly seen in victims of falls, traffic accidents, assaults and homicides (Motherway et al. 2009), but their underlying injury mechanisms can be difficult to elucidate (Wilkins 1997). The fundamental mechanics of cranial bone are still under discussion and by implication, a consensus criterion for skull fracture, which is imperative for the development of intervention strategies to mitigate head injuries, has yet to be established (De Kegel et al. 2019). The difficulties to clarify injury mechanisms in head traumas also introduce predicaments for forensic pathologists, who are commonly faced with the fundamental problem of distinguishing between inflicted and accidental causes (Motherway et al. 2009).

In recent times, Finite Element (FE) models have emerged as useful predictive tools to study injury mechanisms and propose impact tolerance limits for the human head (Giudice et al. 2019). FE models serve as computational surrogates of the human head, and if properly validated prior to utilization, they can simulate dynamic loading responses realistically (Kleiven 2006). Among the many possible applications of FE head models, researchers have demonstrated the potential of FE tools within forensic investigations. For instance, Li et al. (2019) reconstructed suspected infant abuse cases using subject-specific head models and were able to provide biomechanical evidence to support forensic medical evaluations. With a similar scope, Kleiven (2006) reconstructed an injury case involving a woman suffering a lethal skull fracture. Using an FE head model as a prediction tool, Kleiven could dismiss accidental causation of the head injury. Utilizing FE reconstruction in forensic routine practice opens up for objectification of forensic evaluations, avoiding the risk of divergent medical opinions sometimes seen in court cases (Wilkins 1997).

For the above-mentioned applications, precise knowledge of the impact scenario and human subject is required, along with robust and reliable numerical models. A fundamental understanding of the concerning biological tissues is essential, as FE models require accurate material formulations. The human skull, which can be regarded as a three-layered system comprised of two tables of compact cortical bone packing a layer of porous trabecular bone (diploë), is structurally heterogeneous, viscoelastic and has a complex microstructural arrangement. Due to this, modeling failure of cranial bone has proved to be difficult. Further impediments to modeling skull fractures using FE are introduced by the wide variation of input material parameters reported in experimental studies. Stiffness and strength in both diploë and cortical bone vary largely between subjects and with the location of the skull (Boruah et al. 2017; McElhaney et al. 1970). The thickness of the cranial layers (Auperrin et al. 2014), porosity/density (Carter and Hayes 1976, 1977; Melvin et al. 1969) and subject age (Auperrin et al. 2014; Sai et al. 2010) have been identified as probable attributes affecting the fundamental mechanical properties of cranial bone. Most importantly, many researchers have pinpointed morphological variation as one of the key factors to the large spread in reported material parameters (Zhai et al. 2020; Motherway et al. 2009b; Rahmoun et al. 2014).

Various constitutive models for the cranium have been presented in conjunction with FE head models for the purpose of predicting skull fractures (Barbosa et al. 2020; Ren et al. 2020; Cai et al. 2019; Ptak et al. 2018; Lozano-Mínguez et al. 2018; De Kegel et al. 2019; Haut and Wei 2017; Wang et al. 2014). The suggested material models for cranial bone most often encompass isotropic linear-elastic or simpler elastic–plastic formulations combined with brittle material failure laws. The cranial layers in the head models are usually modeled with a fixed thickness ratio in a three-layer system and without subject-specific head geometries. To capture the porosity gradient of cranial bone, researchers are also devoting efforts to establish microstructurally-inspired models (Weerasooriya and Alexander 2021; De Kegel et al. 2019). Recently, De Kegel et al. (2019) implemented subject-specific materials to geometrically personalized skull models to predict skull fractures from cadaveric experiments. In the study, the tensile failure limit and elastic moduli were linked to the locally measured bone mineral density. The FE reconstructions successfully predicted complex skull fractures, demonstrating the promising capabilities of FE models, but also showed difficulties in obtaining precise fracture patterns.

**Fig. 1:**
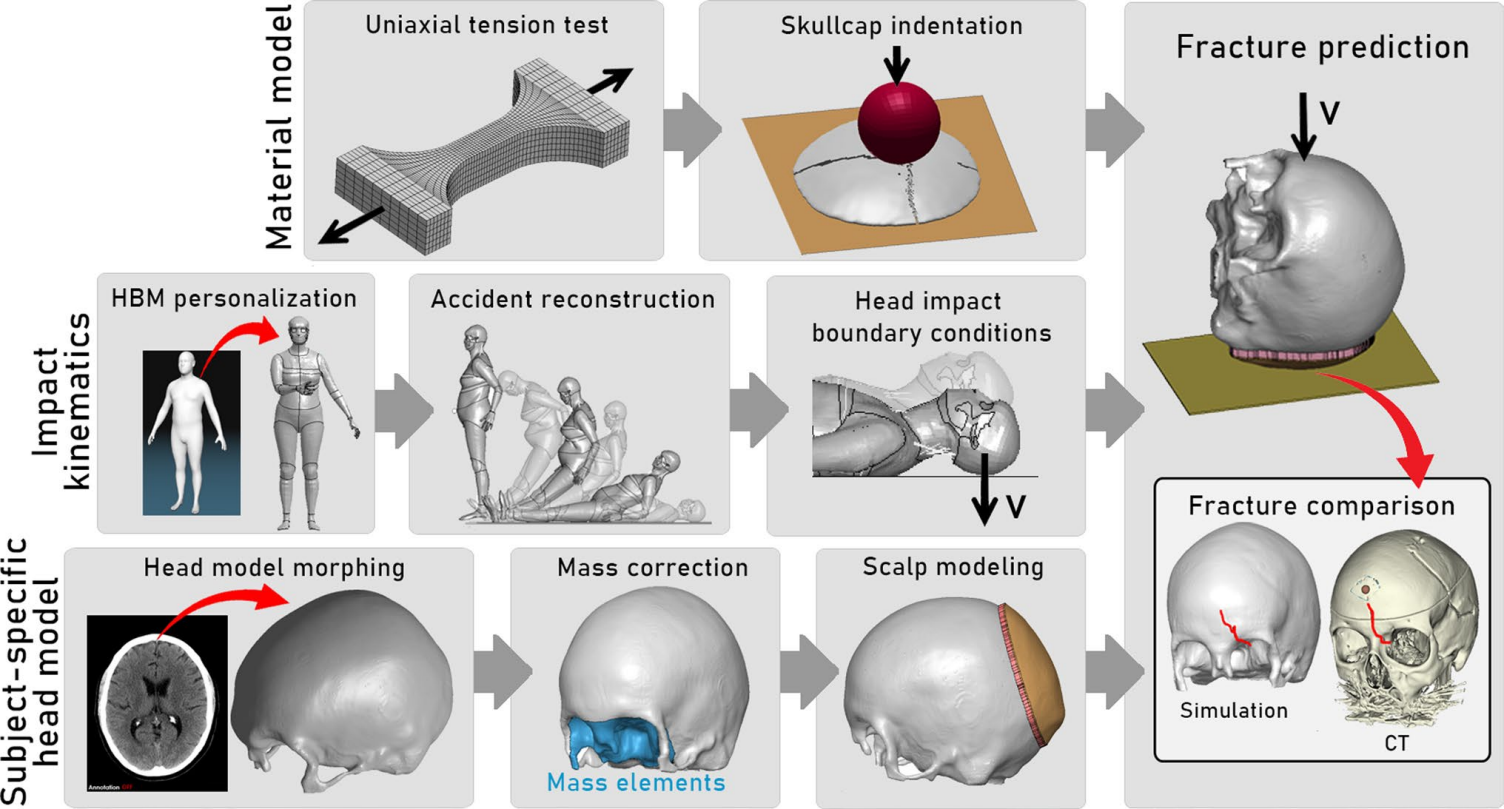
Overview of the study. The material model was established using previously published uniaxial tension and skullcap indentation experiments and subsequently implemented in the macroscale subject-specific head model. Impact kinematics were determined based on accident reconstructions using geometrically personalized HBMs and the forensic records. The subject-specific head model was obtained by following an image-registration-based personalization pipeline, and facial mass and scalp were added before the final simulation setup. Lastly, the simulated fracture lines were compared with segmented PMCT scans of each subject to evaluate the ability of the material model in an FE accident reconstruction pipeline to predict observed fracture patterns

The subject-specific material models are extremely time-consuming and require a complex set of data, usually not obtainable from standard hospital records and medical documentation. In some studies, modeling of the microstructural arrangement of bone and porosity in FE models has shown to have a minimal improvement in the simulation’s predictive capacity (Boruah et al. 2017). And despite researchers emphasizing the viscoelastic properties of cranial bone (Wood 1971; Boruah et al. 2013; Zhai et al. 2020; Motherway et al. 2009b), few strain-rate dependent material models have been implemented in FE head models. Most material models presented in literature use constitutive parameters derived from quasi-static compression or tension tests, even though the simulations are often tested under dynamic loading conditions.

The objective of this paper is to propose a computational approach to characterize the deformational response and fracture of the skull to impact loading by establishing a strain-rate dependent material model for cranial bone, evaluated in two different length scales using experimental data in literature: uniaxial tension of cortical bone specimens (Wood 1971) and skullcap blunt indentation loading (Gunnarsson et al. 2021). With the established material model, subject-specific FE head models were used in reconstructed real-world fall accidents to evaluate the predictive ability of the presented reconstruction methodology. Five forensic fall accident cases were reconstructed and the skull model was assessed based on its capacity of predicting observed skull fracture patterns for these cases. The reconstructions were made with anatomically detailed subject-specific head models, thus incorporating morphological differences among skulls. The procedure to reconstruct occurrences of skull fractures using computational engineering techniques is proposed to be used for accurate skull fracture predictions for various applications and to illuminate the potential of FE as a biomechanically-based tool in evidence-based medicine.

## Materials and methods

An overview of the study scope is provided in Fig. 1. All simulations referred to in this study were preprocessed in LS-PrePost v4.8 and simulated in LS- DYNA v13 (LSTC, Livermore, CA, US) with multiple CPUs and postprocessed in MATLAB v2021a (The 251 MathWorks, Inc., Natick, Massachusetts, United States). All image processing steps, e.g., segmentation and masking of the head Post Mortem Computed Tomography (PMCT) scans, were done using 3D Slicer v4.11 (open-source software available at www.slicer.org).

### Material model development

In this study, cranial bone was modeled in three layers, including an outer and inner cortical table and an in-between layer of diploë. The three-layered structure was modeled with a continuous mesh using solid hexahedral mesh elements. All cranial layers were regarded as structurally isotropic and elastic–plastic. Since the elastic modulus and breaking stress of cranial bone increase with increasing strain rate (Wood 1971), strain-rate dependence was included in the material formulation. The inner and outer cortical bone was assumed to have identical material properties, and the diploë was modeled similarly, only with reduced elastic modulus and failure thresholds. The constitutive model aims at describing the mechanical behavior of cranial bone in dynamic loading events, in the range of small to high tensile strains.

The cranial layers were modeled with a material formulation available in the LS-DYNA library (MAT$\_$187). The model incorporates formulations for strain-rate-dependent plasticity and equivalent plastic strain-dependent failure. Brittle failure was assumed (no damage evolution) and post-failure erosion of elements was implemented. The yield surface of the material is described using a Drucker-Prager cone and failure is described in terms of an equivalent strain failure criterion. For more details of the constitutive model, the reader is referred to the original publications and LS-DYNA manual (Kolling and Haufe 2005; LST 2002).

Tabulated curves for yield stress as a function of plastic strain in tension and compression were used as inputs, as well as a function for Young’s modulus as a function of effective strain rate. The input data for the cortical tables were based on mechanical parameters derived from uniaxial tension experiments (Wood 1971). The elastic modulus *E* [Pa] related to the strain rate $\dot{\varepsilon }$ [1/s] according to

1 $$\begin{aligned} E=(2.32 + 0.28 \log (\dot{\varepsilon }))\cdot 6895 \cdot 10^4, \end{aligned}$$

and the breaking stress $\sigma _B$ [Pa] in tension increased with strain rate $\dot{\varepsilon }$ [1/s] governed by the following relation:

2 $$\begin{aligned} \sigma _B=(11 990 + 1000 \log (\dot{\varepsilon }))\cdot 6895. \end{aligned}$$

Note that the factor 6895 is added to convert the original equation output unit from lb/inch^2^ to Pascal. Equations 1 and 2 stem from statistical analysis of experiments on 120 cortical bone specimens from 30 cadaver heads published by Wood (1971).

The diploë was described using the same material model as for cortical bone, but with stiffness alterations. Using non-contact vibration experiments on dry bone segments harvested from the occipital bone, Kohtanen et al. (2021) reported a 60% lower elastic moduli of the diploë compared to the cortical bone. To model the diploë, the elastic modulus (Eq. 1) was hence scaled with a factor of 0.40 accordingly. Same scaling was done with the shear modulus. Reports on the tensile properties of diploë are scarce, but the quasi-static modulus of elasticity fitted within the reported range for human femur cancellous bone in tension (Carter et al. 1980). Similarly, the failure stress (Eq. 2) was scaled with a factor of 0.10 for the diploë layer quasi-static failure stress to correspond to the lower end of reported breaking stresses of uniaxial quasi-static tension for 22 human femur specimens (Carter et al. 1980).

The elastic moduli of the cranial layers were assumed to be the same in tension and compression. The assumption is based on experimental findings (Oftadeh et al. 2015; Robbins and Wood 1969). A tensile strain failure criterion was specified and elements were not allowed to break in compression. Other material parameter inputs are presented in Table 1.

**Table 1:** Material parameters of the scalp (Fahlstedt et al. 2015; Li et al. 2017) and cranium. The bone densities are based on reported experiments by Kohtanen et al. (2021) and the shear modulus for cortical bone is based on work by Tang et al. (2015). Poisson’s ratio for cortical bone and diploë was based on Peterson and Peterson and Dechow (2002) work and McElhaney et al. (1970) respectively

	Density	Poisson’s ratio	Shear modulus	1st shear modulus	1st exponent
	$\rho$	*v*	*G*	$\mu _1$	$\alpha _1$
	[kg/$\hbox {m}^{3}$]	[−]	[Pa]	[Pa]	[-]
Cortical bone	2048	0.20	$2.90\cdot 10^9$	–	–
Diploë	1290	0.20	$1.16\cdot 10^9$ (scaled)	–	–
Scalp outer	1130	0.50	–	120000	24.2
Scalp inner	1130	$\sim$0.50	–	3990	8.82

#### Evaluation against experimental data from literature

The material model for cortical bone was evaluated against experiments in uniaxial tension conducted by Wood (1971). Wood extracted dogbone-shaped coupons of cortical bone from human cadaver skulls and subjected them to quasi-static and dynamic loading. In the present study, dogbone-shaped coupons with the same dimensions as presented by Wood were modeled using $\sim$4000 hexahedral elements. The element size was comparable to the mesh element size in the subject-specific head model (see Sect. 2.2.2). Displacement was prescribed to one end of the coupon to obtain strain rates of 0.1/s, 10 /s and 150/s. A fixed boundary condition was applied to the opposite end. The specimen was loaded until catastrophic failure. The simulated stress–strain curves were ultimately compared with the published ex vivo stress–strain curves (Wood 1971).

**Fig. 2:**
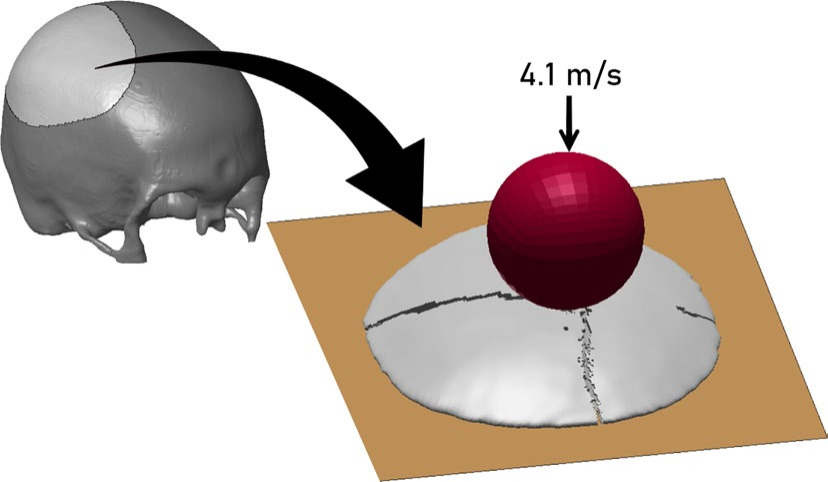
Skullcap blunt indentation simulation of Gunnarsson et al. (2021) experiment

Skullcap specimens are larger than the conventional beam strips, circular coupons or dogbone-shaped specimens usually seen in mechanical testing of skull bone, yet they enable more tractable experiments than full-size cadaver heads and capture the multiaxial stress state of cranial bone. To demonstrate the predictive abilities of the constitutive model and visualize its fracture response, a blunt indentation of a skullcap model was simulated, see the procedure in Fig. 2.

The simulation setup was designed to follow the experiments conducted by Gunnarsson et al. (2021). In the experimental study, dome-shaped specimens were cut from the upper part of cadaver skulls, excluding any sutures, with an outer diameter between Ø75-150 mm. The skullcaps were indented using a hemispherical indenter tip of Ø31.75 mm, impacting the specimen on the outer cortical bone. The indenter coincided with the center of the inner cortical surface. The specimen rested freely on a metallic plate.

In this study, a skullcap specimen was extracted from the parietal bone of one of the subject-specific head models (later presented in Sect. 2.2.2). The skullcap was meshed with $\sim$150,000 solid hexahedral elements using the software Hexotic. The semi-major axis length was approximately 78 mm across. The skullcap was indented with a velocity of 4.1 m/s for the duration of 300 $\mathrm {\mu }$s. The indenter tip and metal support plate were modeled as rigid, and the friction coefficients were set to 0.1. The plate was constrained in all directions, while the indenter was restricted in the directions tangential to the bottom support plate. The mode of failure, fracture lines, as well as force-displacement response was then compared with the ex vivo observations (Gunnarsson et al. 2021).

**Table 2:** Descriptions of cases and subjects. The subjects’ age, height and weight are withheld to maintain subject integrity

	Sex	Age [years]	Height [cm]	Weight [kg]	Accident description	Impact surface	Impact site
Case 1	M	$>60$	$>170$	$<70$	Fall backwards from standing	Tile	Occipital bone
Case 2	F	$>60$	$<170$	$>70$	Fall backwards from standing, lands on buttocks, left elbow braces fall. Fall induced by impact in the abdomen by bicycle traveling at 25 km/h	Asphalt	Occipital bone
Case 3	M	$>60$	$<170$	$>70$	Fall backwards from standing	Tile	Occipital bone
Case 4	M	$>60$	$<170$	$<70$	Fall backwards from standing	Dry soil	Occipital bone
Case 5	F	$>60$	$<170$	$>70$	140 cm vertical drop. Lands prone with knees, shins on lower stair steps, lands on arm in front of chest	Tile	Frontal bone

### Description of forensic cases

Five real-world fall forensic fall cases (denominated Case 1 to 5) were selected from a database of deceased persons autopsied at the Department of Forensic Medicine at the University of Copenhagen (Henningsen et al. 2022). All subjects suffered blunt force skull trauma, each documented with Post-Mortem Computer Tomography (PMCT) of the head. The events of the accident were delineated and impact points were traced by an experienced forensic pathologist and a junior doctor. Conclusions were based on police and autopsy reports, hospital records and PMCT. The PMCT were segmented, excluding the facial bone and facial soft tissues. The scalp, outer and inner cortical bone, and diploë were separated in layers by masking. The diploë and cortical bone were separated in the segmentation by their CT-value (Hounsfield Unit). The entire skull thickness made up one single segmentation. Within that segmentation, the segmentation for the lower HU diploë was subsequently made with the “thresholding tool” in 3D Slicer and set to overwrite the cortical bone segmentation (that of the entire skull thickness). The scalp was merged into a single layer combining the dense connective tissue scalp and soft tissue scalp. The masks were created using a semi-automated approach in 3D slicer (Fedorov et al. 2012).

The five forensic cases were either falls from standing, falls from heights or low-speed traffic accidents. Four forensic cases concerned backwards falls, impacting the occipital bone. One case involves a fall from height, leading to a frontal bone impact. The details of the forensic cases are presented in Table 2. In Cases 1 to 4, the subject obtained a linear skull fracture in the occipital bone, while in Case 5, the subject obtained a circular fracture in the frontal bone with a linear fracture radiating down and extending through the orbital roof. Whether the mid or lower part of the face struck the ground first was not evident.

#### Accident reconstructions using HBMs

To obtain the head impact velocities of the fall accidents, all five cases were reconstructed using geometrically personalized human body models (HBMs) (see Fig. 4). An upright 50^th^ percentile pedestrian version of the SAFER THUMS HBM v10 (Pipkorn et al. 2022) constituted as the baseline HBM, which was subsequently nonlinearly morphed to match the anthropometry of the subjects. The external surface (skin) of the HBMs was morphed to a standing male or female reference geometry with the same height, age and body mass index (BMI) as the case subjects. The reference geometries were from open-source, originally developed from statistical analyses of high-resolution laser scans and anthropometric measurement data of human subjects (Humanshape.org, 2022). The density of the soft tissues of the HBM was adjusted to obtain the correct mass of the case subjects. The morphing procedure followed the same essential steps as presented by Li (2021). The reader is referred to the original publications for further details.

**Fig. 3:**
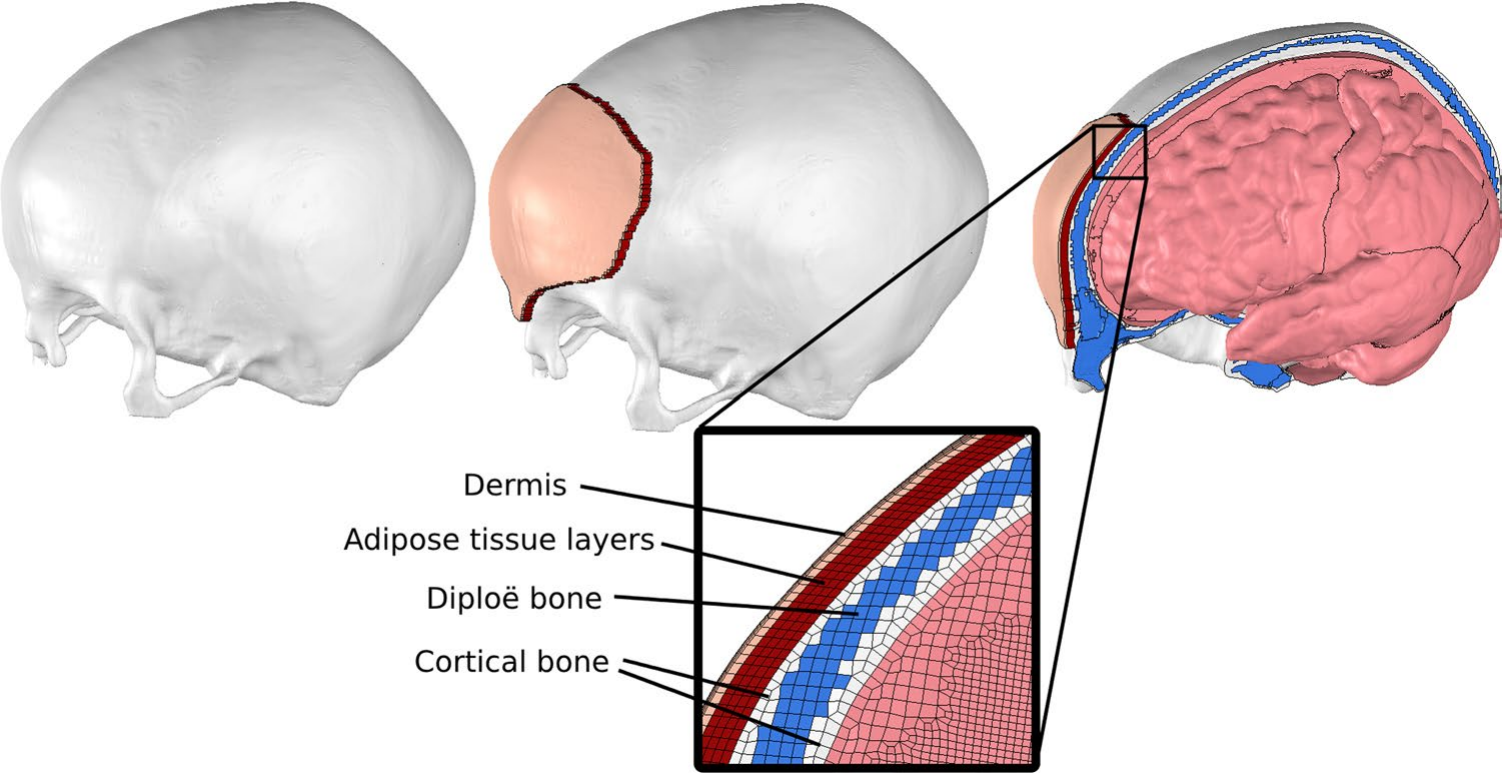
Head model assembly with section- and detailed view of the cranial bone and its layers

**Fig. 4:**
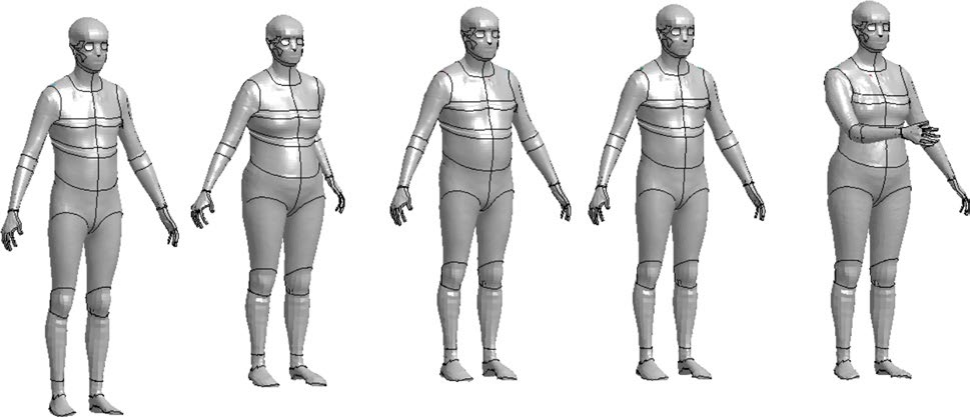
Personalized HBMs for Case 1 to 5 (left to right)

The approximate location, orientation and leg/arm positions were based on forensic assessments. The HBMs were positioned using the marionette method, partly described by Poulard et al. (2015), where a cable between node pairs was applied to move the model into the desired position. The cables (formulated as beam elements) were defined with an initial tensile force that forced the movement, and displacement dampers were used to help the model settle during simulation. For Case 2, the left elbow was positioned to brace the backwards fall. For Case 5, the right arm was positioned in front of the HBM’s chest.

To reconstruct Cases 1, 3 and 4, the HBMs were positioned with an initial $15^{\circ }$lean and with applied gravity load. No initial velocity was applied. The knee joints were locked to reduce degrees of freedom and avoid inconsistent results.

For the reconstruction of Case 2, a cylinder resembling bicycle handlebars was modeled as an impactor. The cylinder was given an initial velocity of 25 km/h for 30 ms, impacting the HBM frontally in the abdomen. The HBM was given no initial lean nor velocity, and no joints were locked.

For Case 5, the HBM was positioned almost horizontally at a height of 140 cm with an applied gravity load. An initial lean was applied to assure that the HBM landed prone with knees on the lower stair steps. An extra ground surface was added to resemble the lower staircase steps where the shins impacted.

After simulation, the head velocity at the time step prior to head impact was extracted (from the head center of gravity) and applied as boundary conditions in the subject-specific FE head simulation.

#### Head impact reconstruction using subject-specific head models

The boundary conditions, i.e., the head impact velocities, obtained from the HBM simulations were applied to subject-specific head models in order to predict respective skull fractures. The head models were positioned to initially strike the impact points derived by forensic investigation for each subject. In contrast to the head of the HBMs, the subject-specific head models were constructed with a fine enough mesh to distinguish fracture lines and have more anatomical detail compared to the generic HBM head model.

The subject-specific head models were developed based on a baseline head model (ADAPT) (Li et al. 2021; Li 2021), which includes the brain, meninges, cerebrospinal fluid, superior sagittal sinus and the skull. The skull of the ADAPT model has been divided into the inner cortical bone, the porous diploë and the outer cortical bone, as illustrated in Fig. 3. The model is meshed with a continuous mesh of about 4 million hexahedral and 0.50 million quad elements. The element size ranged from 0.50 mm to 2.50 mm. The model has been evaluated against experimental data of brain-skull relative motion, brain strain and intracranial pressure. The ADAPT model, along with its diploë-cortical bone ratios, was originally developed based on reconstructions of the generic ICBM152 template generated from MRI scans of 152 healthy adult subjects (Fonov et al. 2009).

The baseline ADAPT model was morphed for each case following an image-registration-based personalization pipeline established by Li (2021). The approach includes image registration procedures, mesh morphing and mesh grouping. PMCT scans were segmented into scalp, skull, and cranium for each of the five subjects. The segmentations were then rigidly aligned to the ADAPT head model. The segmented PMCT scans and corresponding masks from each of the five subjects served as templates, or so-called fixed images, for the morphing procedure. A displacement field was obtained using image registration and was used to morph the ADAPT to five subject-specific head models, capturing the subject’s skull geometry. The morphing was done using a Demons registration algorithm implemented in the 3D Slicer module BRAINSdemonWarp (Johnson and Zhao 2009). Using the proposed method, the thickness, geometry and size of the FE skull remain true to the subject’s PMCT. As the inner and outer skull surfaces were morphed, the original diploë-cortical bone thickness ratio was transformed in consonance with the applied displacement field. In some regions, the diploë is more announced, while it is almost non-existent in others. Only a brief overview of the morphing procedure and head model is provided here; additional details have previously been documented (Li et al. 2021; Li 2021).

To quantify the personalization accuracy, Dice scores were calculated. Dice is a measurement of the spatial overlap between the subject-specific head models and PMCT scans (Ou et al. 2014). For the five personalized head models, the average Dice scores were all >0.80. A Dice value of 0 implies no overlap of the PMCT segmented surface and the morphed surface, while a Dice value of 1 implies a perfect overlap. The minimum Jacobian of respective subject-specific head models ranged between 0.12$-$0.31.

As the ADAPT head model does not include the scalp, a locally applied patch of the scalp was added in the model, covering the impacting region of the skull. The scalp was divided into two layers, with one layer representing the dermis and the second representing the two adipose layers of the scalp and the intermediated galea aponeurotica. Both layers were modeled with a first-order Ogden hyperplastic model (*MAT$\_$077$\_$O in the LS-DYNA library) with the parameters presented in Table 1. The scalp was uniformly thick with six layers of solid elements in the skull’s outer surface’s normal direction, as seen in Fig. 3. The thickness of each subject’s scalp was based on measurements in CT scans at the impact site. By using the locally applied scalp instead of a scalp covering the whole skull, the ADAPT model spares up to a million elements. The friction coefficient between the scalp and impacting material was assumed to be 0.40, which is within the reported range for human skin (Derler and Gerhardt 2012).

Additional nodal mass was equally distributed to a node set covering the orbital roof and skull base of the FE head model to account for the missing facial mass. With facial tissues and facial bone, the head was assumed to have a total weight of 4.03 kg (Yoganandan et al. 2009). The added mass was hence equal to the difference between 4.03 kg and the mass of the FE model measured without the lumped mass elements.

The impacting material of all cases was assumed to be relatively stiff, in line with tile, asphalt, concrete and hardpacked soil. The impacting surface was modeled as a linear-elastic material, with an elastic modulus of 10 GPa, Poisson’s ratio of 0.25 and density of 2700 kg/m^3^.

Through-thickness fracture lines were inferred as areas where mesh elements had eroded in all three cranial layers. The duration time of the simulation was 6.0 ms, covering the whole impact event.

## Results

**Fig. 5:**
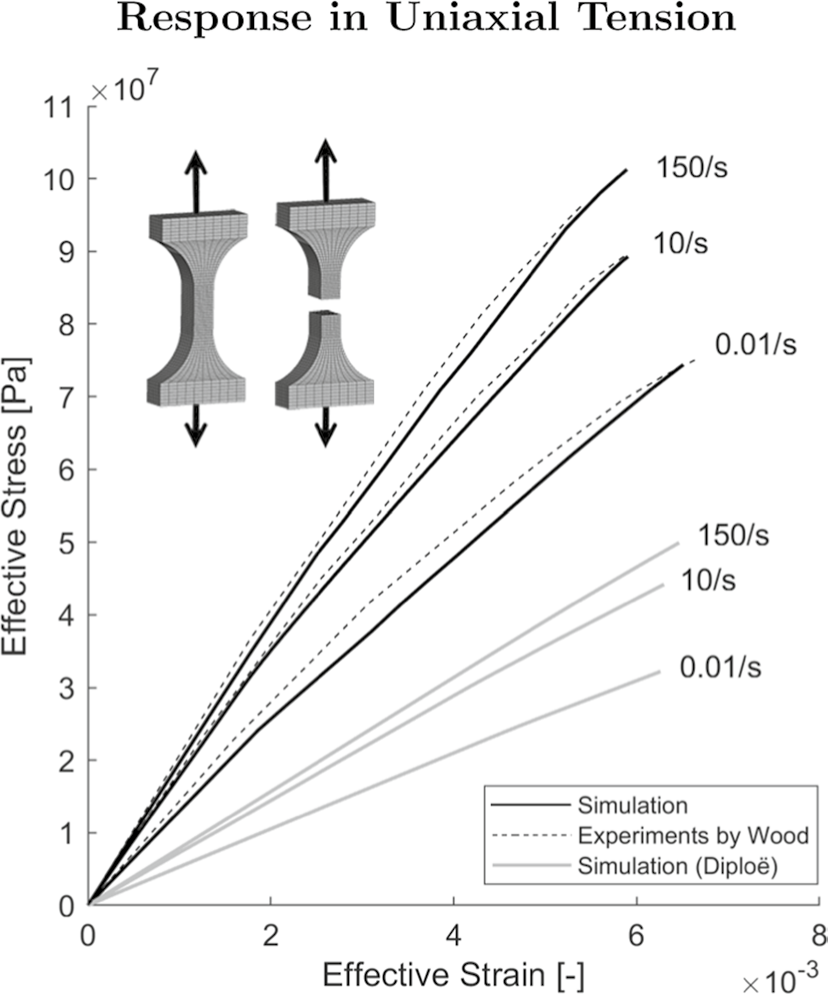
Experimental (Wood 1971)) and simulated stress–strain curves for dogbone-shaped cortical bone specimen tests in tension plotted until failure for three strain rates. After failure, the stress in the elements is equal to zero. Tension tests for diploë are added for reference

**Fig. 6:**
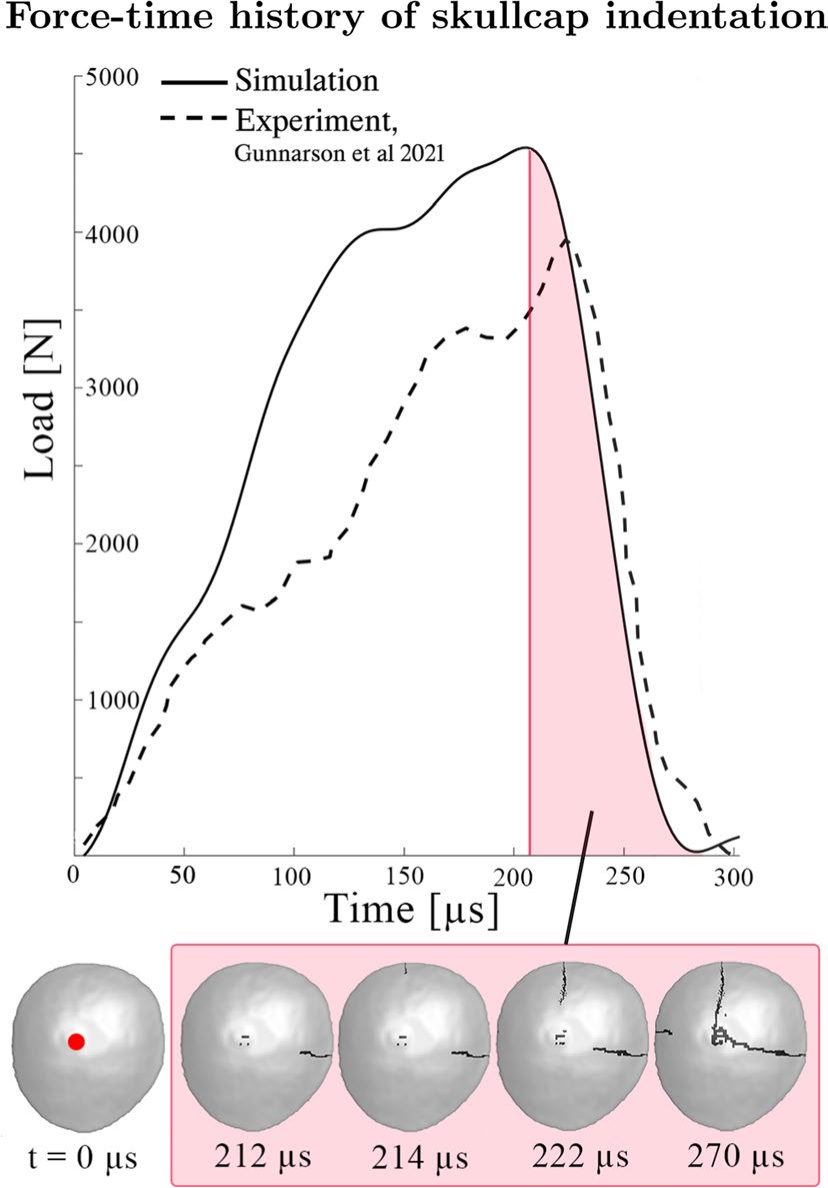
Experimental (Gunnarsson et al. 2021) and simulated force applied to skullcap specimen, with illustrated fracture initiation and propagation prior to catastrophic failure. The specimen is seen from above, viewing the outer cortical bone. The impact point is shown in red

### Performance of cranial material model

The experimental (Wood 1971) and simulated stress–strain curves for the dogbone-shaped specimen in uniaxial tension under three different strain rates are presented in Fig. 5. One single fracture was initiated perpendicular to loading in the middle of the specimen and quickly resulted in the failure of all elements in the mid-region. All elements in the mid-cross section plane eroded and no necking occurred.

In Fig. 6, the experimental (Gunnarsson et al. 2021) and simulated force-time histories of the indenter in the skullcap simulation is presented together with an illustrated timeline of the fracture propagation. The first crack was initiated in the center of the inner cortical table and propagated toward the edge of the skullcap to the diploë and outer cortical table. The crack grew into a through-thickness crack from the skullcap edge and propagated toward the impact point at the center region. Before reaching the impact region, a through-thickness secondary crack was initiated from the specimen edge. It traveled toward the impact point in the same manner as the initial crack. The final catastrophic failure occurred when the two cracks intersected at the impact region. Lastly, two smaller cracks were initiated. One originated from the impact region and radiated away from it, and a third crack was initiated at the opposite edge of the first crack. The load in the skullcap simulations was measured as the reaction force of the indenter in the load direction and reached a maximum of 4540 N. Macro-fracture initiation occurred after peak force was reached.

### Head impact boundary conditions

**Fig. 7:**
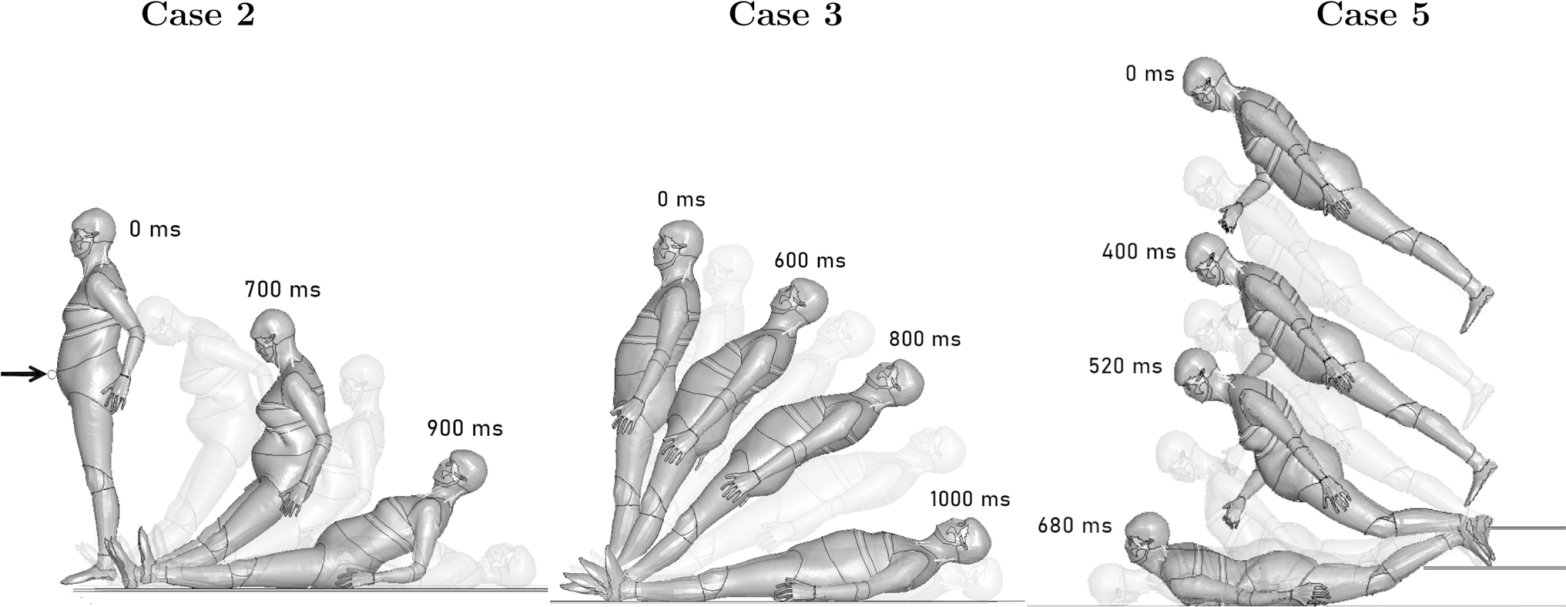
Fall sequence of reconstructed fall accidents plotted at a selection of time steps. Note that Cases 1 and 4 have almost identical fall time-histories as Case 3

The fall sequences of the reconstructed cases using personalized HBMs are illustrated in Fig. 7. Note that Cases 1 and 4 have almost identical fall time-histories as Case 3. The obtained head impact velocities, along with the measured scalp thicknessess and applied facial mass, are presented in Table 3. The head velocities are presented component-wise: the velocity component transverse to the ground and the velocity component normal to the ground.

**Table 3:** Measured scalp thicknesses and added facial masses for each subject, as well as the applied head impact velocities that were derived using geometrically personalized HBMs. *[2-column fitting table]*

	Scalp thickness	Added facial mass	Transverse velocity	Normal velocity
	[mm]	[kg]	[m/s]	[m/s]
Case 1	8.08	1.16	1.07	$-$5.46
Case 2	5.76	0.92	13.8	$-$3.62
Case 3	5.29	0.83	0.80	$-$5.63
Case 4	7.43	1.18	0.97	$-$5.58
Case 5	4.47	1.07	0.00	$-$4.45

### Predicted skull fractures

**Fig. 8:**
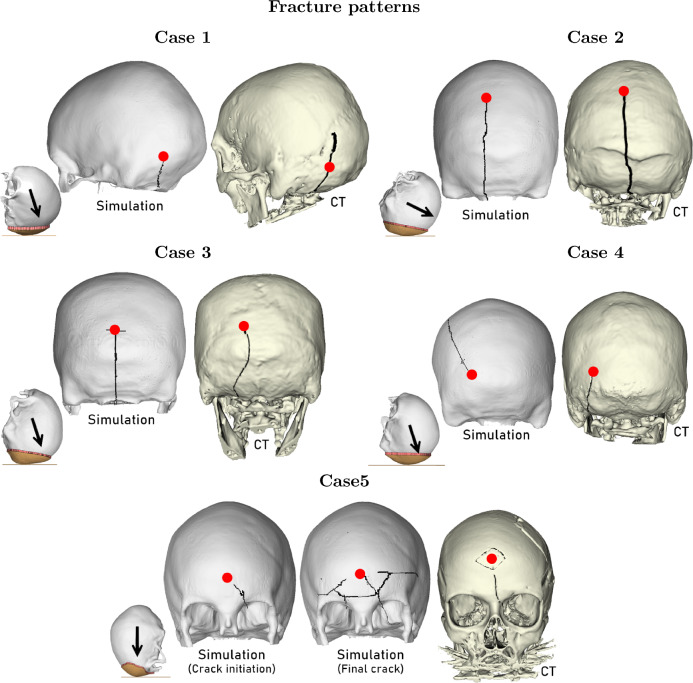
Final fracture patterns of reconstructed Cases 1 to 5. The initial head positions in the simulations are illustrated with arrows to indicate the resultant velocity directions. Attested impact locations are highlighted in red and the fracture lines are illustrated in black. Note that the subject in Case 5 went through a craniotomy, hence some additional lines are visible. The craniotomy and segmentation artefacts are not to be confused with fracture lines resulting from impact hued in black. The visualization of the actual fracture lines is also affected by partial healing. Cases 2 to 4 are presented in a posterior view, Case 1 is shown in a posterior-sinister view, and Case 5 is presented anteriorly

**Fig. 9:**
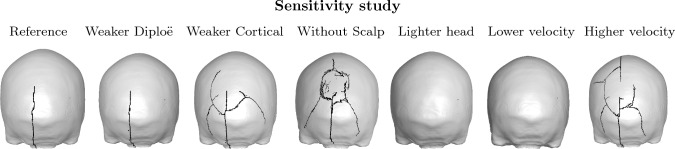
Sensitivity study by investigation of five simulation alterations. Case 2 was simulated once with weaker diploë and cortical bone, once without the scalp, once with a lighter head mass and once with lower and higher normal head impact velocity

The final skull fractures are presented in Fig. 8. In all simulations, fracture was initiated at a distance away from the impact region, and almost instantaneously propagated toward and/or away from the impact site. All visualized fracture lines were through-thickness fractures. Each through-thickness fracture was initiated with eroded elements in the inner cortical table.

In Case 1, the simulated fracture follows a path toward the foramen magnum, similar to what is seen in PMCT. The fracture line was not predicted to run superiorly-anteriorly toward the lambdoid suture by the FE simulation.

In Case 2, an anteriorly-posteriorly running linear fracture was predicted. The fracture line extended to the foramen magnum, analogous to the fracture seen in PMCT.

In Case 3, a T-shaped fracture with a vertical leg propagating toward the foramen magnum was predicted. The horizontal leg was not observed in PMCT, but the location and nature of the linear fracture are comparable. The fracture in PMCT follows a path more curved than predicted by FE. The FE model further predicts a fracture propagation across the foramen magnum onto the sphenoid bone. No such extension of the fracture was seen in PMCT.

In Case 4, the actual fracture seen in PMCT extended inferiorly from the impact site in the occipital bone toward the foramen magnum. The simulation predicted a fracture progressing anteriorly-superiorly in the parietal bone.

In Case 5, the FE simulation overpredicted the skull fracture pattern seen in PMCT. In the FE model, an initial fracture was found close to the orbital rim, extending toward the impact point. The final crack pattern consisted of several fracture lines radiating from the impact point, and one encircling fracture line, concentric to the ground contact area. Both initial and final comminuted fracture patterns are illustrated for Case 5 (Fig. 8). Similarities of the rhomboid shape fracture with the linear fracture extending into the left orbital and anterior fossa can be distinguished.

### Sensitivity study

A sensitivity analysis was performed by simulating Case 2 with modified simulation conditions. The following was investigated:

1. Reduced diploë strength by reducing the diploë ultimate tensile strain by $\sim$0.001 (see Appendix).
2. Reduced cortical strength by reducing the cortical bone ultimate tensile strain by $\sim$0.001 (see Appendix).
3. Complete removal of scalp
4. Reduced head mass by 0.50 kg
5. Reduced impact normal velocity by 0.50 m/s
6. Increased impact normal velocity by 0.50 m/s

The resulting fracture lines from the test (a)–(f) are presented in Fig. 9. The magnitude of the fracture line was unchanged by reducing the diploë failure strain. The fracture grew more severe in Case 2 by reducing the cortical bone strength. Similarly, by removing the scalp, additional linear fractures radiating from the apparent central region occurred. A concentric fracture that was intercepted by the radiating arms of the fracture was also predicted by the FE model. The impact did not result in any through-thickness fracture lines by reducing the head mass or impact velocity by 0.50 kg and 0.50 m/s respectively. A linear fracture line in the inner cortical table (similar to the reference fracture line) was observed in the simulation with reduced head mass. Increasing the normal velocity by 0.50 m/s also magnified the fracture severity. Additional investigated parameters sensitivities are found in the Appendix.

## Discussion

This study provides a currently lacking strain-rate dependent material model of cranial bone that has the capacity to accurately predict linear fracture patterns. Linear skull fracture, which is the most common fracture type (Asirdizer et al. 2021; Whitwell et al. 2021), are believed to originate from tensile stresses induced by an outward bending of the cranium at a distance of the impact location (Gurdjian et al. 1949). Initiated by the outbending, a linear fracture usually extends to the impact point as well as in the opposite direction toward regions of structural weakness, e.g., the skull base (Whitwell et al. 2021). A novel procedure to reconstruct occurrences of skull fractures, capturing this all-in-all fracture initiation, propagation and final pattern, using computational engineering techniques is presented.

### Mechanical response

The model has been shown to reproduce the mechanical response of cortical bone in tension, while correctly capturing failure mechanisms in blunt indentation loading of a skullcap specimen. The computationally derived stress–strain curves presented in this study were consistent with the experimental findings on cortical bone (Wood 1971). The cortical bone specimens in ex vivo experiments all failed in a plane approximately perpendicular to the long axis. In agreement, perpendicular catastrophic fractures were observed in the simulated tension tests.

In the simulated blunt indentation loading of a skullcap specimen, the skullcap broke into two separate pieces. Two through-thickness linear fractures were initiated at the skullcap edge and propagated toward the loading point. The intersecting fracture lines were almost perpendicular to each other, creating a nearly $90^{\circ }$circle sector (see Fig. 6). This has noteworthy similarities to the corresponding experiments (Gunnarsson et al. 2021), incorporating two conducted high-rate loading ex vivo experiments. In the first experiment (impact velocity of 4.1 m/s), a specimen was loaded until through-thickness cracking split the skullcap into two separate pieces. In the second (impact velocity of 3.8 m/s), the specimen was unloaded before macro-fracture was initiated. The primary macro-fracture initiation location was at the specimen edge for both specimens, agreeing with the in silico results presented in this study. In the first ex vivo specimen, the initial edge fracture subsequently propagated through the loading point. A second macro-fracture was initiated at the impact point and propagated to the edge, resulting in a fracture line almost perpendicular to the first fracture line. The two linear fractures intersected at the loading point and radiated toward the edges in one direction, cutting the skullcap into two pieces (one $270^{\circ }$ circle sector and one $90^{\circ }$ circle sector), similar to the in silico catastrophic failure presented in Fig. 6.

In the in silico skullcap loading test, two edge cracks were initiated before catastrophic failure. In accordance, two edge micro-fractures approximately one-third of the loading point to edge distance were observed in the second ex vivo specimen. In the second ex vivo experiment, a small micro-fracture directly under the loading point was observed before the macro-fracture was initiated, as seen in silico. Gunnarsson et al. (2021) also observed micro-fracture initiation in the inner table just at or near the loading point. Before macro-fracture in the simulations, micro-fracture in the inner table was documented. The inner table micro-fracture initiated right below the impact point.

The peak force observed in the blunt indentation loading experiments was measured to be $\sim$4.5 kN in silico and $\sim$4.0 kN ex vivo, suggesting that the stiffness of the in silico model is overestimated. The differences in fracture lines and peak force can be attributed to the difference in the morphology of the skullcaps. The skullcaps for the experiments and simulations are not taken from the same skull specimens, and thus have incongruent diploë-cortical bone ratio, shapes, and sizes. As stated previously, morphological variation is usually argued to be a key contributor to mechanical variations in experiments (Zhai et al. 2020; Motherway et al. 2009b; Rahmoun et al. 2014). Regardless, the model shows good failure mode prediction compared to the experiments: the in silico skullcap did experience load increase, followed by brief softening and load plateau, before macro-fracture initiation.

### Accident reconstructions using HBMs

The head impact velocities of the simple backwards fall case (Case 1, 3 and 4) showed little variation. The normal velocity component ranged between 5.46 to 5.63 m/s for the three cases. Hamel et al. (2013) performed rigid body simulations in comparable backward falls with body models that were scaled according to combinations of three body heights (160, 170 and 180 cm) and two body masses (50 and 75 kg). As in the present study, the body was applied an initial lean and without an initial velocity. The joints were simulated two times, once with locked joints and once with unlocked joints. In disagreement with this study, Hamel et al. showed a larger variation among subjects: an increase in head impact velocities with subject height and subject weight was observed. However, looking at all scenarios, the head impact velocity ranged between 3.71$-$5.02 m/s (locked joints) and 4.62$-$6.15 m/s (free joints). The impact velocities reported using FE HBMs in this study were close to the reported range. It is worth noting, that other head impact velocities have been reported using test dummies. Hajiaghamemar et al. (Hajiaghamemar et al. 2015) reported lower head impact velocities looking at backward fall using 50th percentile male test dummies. The translational head impact velocity for falls with and without hip flexion was derived to be 4.85 m/s and 6.75 m/s respectively.

### Skull fracture prediction capacity

The FE model did predict the actual occurrence and extent of skull fractures in all cases. However, the fracture patterns were predicted with varying levels of agreement with fractures seen in PMCT scans.

In Case 4, the FE model suggests that the fall induced a linear skull fracture progressing anteriorly-superiorly from the impact site on the occipital bone. The actual fracture seen in PMCT extended inferiorly from the impact site in the occipital bone toward the foramen magnum. The simulation-PMCT discrepancy can have several possible explanations. Some might lie in the morphology of the skull, and some in the assumptions and simplifications made. This particular case is further discussed in the Appendix. Regardless, it should be emphasized, that even though the fracture does not correspond to the real-world fracture, the FE head model does in fact accurately predict the actual occurrence of a linear skull fracture. Case 4 simulation thus provide valuable information about the possible mode of injury, even though the fracture propagation might be questionable. Since propagation and initiation of skull fracture have previously not been accounted for in previous published fracture prediction attempts, the results of this study are especially noteworthy. Case 4 also emphasizes how sensitive the model is to input parameters. The same remark was highlighted by De Kegel et al. (2019) in another attempt to predict experimental skull fracture patterns using subject-specific FE models. The authors raised concerns about the robustness of FE models for the application, as they observed considerable sensitivity to modeling parameters (e.g., impact point and geometry).

The FE model overestimated the frontal skull fracture in Case 5. In this case, the overestimated fracture might be coupled with an overestimated impact velocity. In general, one can assume that the more severe the fracture, the greater the impact force, and with increasing force comes additional linear fractures at secondary or tertiary stress areas (Whitwell et al. 2021). Out of the five accidents, Case 5 is associated with the most unknown parameters. Firstly, despite extensive forensic investigation, the initial point of impact, whether it was the face or forehead, was not concluded. Secondly, the subject falls with the right arm in front of the chest, resulting in a broken humerus. It is unknown to what extent the subject braced the fall, and the arm might have dampened the fall more than indicated by the HBM simulations. Such events are challenging to capture using HBMs without muscle activation. In other words, the Case 5 head impact velocity includes a level of uncertainty. As highlighted by the sensitivity study, the model is very sensitive to the applied impact velocity.

In Case 3, an occipital bone fracture was accurately predicted. Yet, the simulated fracture line continued to propagate across the foramen magnum (the entry point of the spinal cord that pierces the occipital bone) and extended into the anterior fossa. Although it is not uncommon that fractures in the occipital bone extend to the posterior and anterior fossa (Whitwell et al. 2021), no basilar fracture was observed in PMCT. An explanation for the overpredicted fracture line might be found in the skull mesh artefacts. The Dice values were the lowest in the region surrounding the foramen magnum. Meaning, the model geometry is the least accurate in that particular area compared to the rest of the head. Also, the mesh at the edges of the foramen magnum is relatively sharp and jagged. Such abrupt changes in geometry could lead to stress concentrations, which might possibly allow fracture lines to erroneously travel across the foramen magnum as seen in Case 3.

### Validity of material model

The tensile properties of diploë in dynamic loading are not as widely reported as cortical bone and for that reason, modeling simplifications were made throughout this study. One noteworthy assumption was the less predominant role of the diploë in the mechanical response of skull bone. The diploë was assumed to merely transfer mechanical loads from the outer cortical surface to the inner, unlike the cortical tables that were assumed to have the primary load-carrying functions. This premise, that the diploë had less of an effect on the structural integrity of the skull compared to cortical bone, was based on fundamental theories by Wood (1971) and related experimental findings (Zhai et al. 2020; Melvin et al. 1969). Under this assumption, the diploë was given less attention in the constitutive modeling. Essentially, the diploë was assumed to act just like cortical bone, only weaker in respect of elastic modulus and ultimate tensile strength. In reality, diploë can be argued to differ substantially from the cortical bone in a mechanical sense. To support the made assumption, Case 2 was simulated once with a lower tensile strength of the diploë and once with a lower tensile strength of the cortical bone. By comparing the simulations with the original Case 2 simulation, the decrease in diploë tensile strength had a negligible effect on the final skull fracture. The decrease in cortical strength on the other hand, had an obvious influence on the predicted fracture. The sensitivity analysis strengthens the hypothesis that the constitutive modeling of cortical bone is of more importance than the diploë for the current application. Of course, the mechanical behavior of the diploë should not be completely disregarded and is left as an objective for future studies.

The focal point of this study has been the tensile failure of cranial bone and compressive failure has been somewhat disregarded. The rationale behind this approach is the fact that linear impact-induced skull fractures are likely to arise from tensile stresses in the skull near the impact site (Wood 1971; Melvin et al. 1969). Also, bone is known to have a greater tolerance to compressive forces than tensile (Whitwell et al. 2021) and the compressive failure strength of cortical bone is considerably higher than the tensile failure strength (Cezayirlioglu et al. 1985; Yeni et al. 2004). It can thus be argued, that the tensile properties of bone are of more relevance than the compressive properties in blunt force traumas of the nature presented in this study. The approach has been followed in previous studies, and excluding compressive failure has been shown to be a successful strategy to obtain linear fracture patterns (De Kegel et al. 2019).

The general material model for cranial bone presented in this study includes additional simplifications that could be taken into consideration in future studies. Firstly, the material parameters were the same for the inner and outer cortical bone in this study, even though researchers have argued that the stiffness in the two layers differs. Peterson and Dechow (2002) determined the elastic properties of human parietal cortices ultrasonically and argued that the outer table is stiffer than the inner. Cortical bone was also suggestively anisotropic, contradictory to the results of McElhaney et al. (1970) and Wood (1971), arguing for tangential isotropy. Secondly, the regional variation of mechanical parameters that has been suggested by numerous studies (Boruah et al. 2017; McElhaney et al. 1970; Kohtanen et al. 2021) have not been accounted for in this study. It should be mentioned that some researchers have also claimed no regional variance (Wood 1971). Thirdly, the proposed model does not include any consideration of the subject’s age, which has been claimed to also be a determinant of the mechanical parameters (Auperrin et al. 2014; Sai et al. 2010). Here too, Wood (1971) did not find any age-dependence in breaking stress or strain of cortical bone in uniaxial tension, and the age-dependence of the elastic modulus was found to be very small. Lastly, the human cranial bones are connected with each other by fibrous joints (sutures). The cranial sutures were not taken into consideration in this study and their effect on the fracture pattern should be investigated.

The proposed model includes several advantages. First of all, the material formulation is unusually general in relation to its decent predictive ability. Lately, subject-specific material models have been seen in literature (De Kegel et al. 2019; Weerasooriya and Alexander 2021). These types of models require ample pre-processing and computation time, and have been implied to lead to minimal improvement in fracture predictability compared to non-subject-specific models (Boruah et al. 2017). The material model suggested for cranial bone in this study requires a limited amount of independent material properties and demonstrates the potential of utilizing general material formulations. Secondly, the proposed material model could be further advanced easily by, e.g., implementing experimental data for shearing and a wider selection of strain rates, or other input supported by the material formulation *MAT$\_$187 available in the LS-DYNA library. Subject- and region-specific tuning of breaking stress and elasticity moduli might improve the predictive outcome of the model.

## Conclusions and future work

A novel procedure to reconstruct occurrences of skull fractures using computational engineering techniques, to capture the all-in-all fracture initiation, propagation and final pattern, has been presented. An isotopic, strain-rate dependent and elastic–plastic material model for cranial bone has been proposed and evaluated in a set of selected applications. The model has been shown to reproduce the mechanical response of cortical bone in tension, while correctly capturing failure mechanisms of a linear fracture in blunt indentation loading of a skullcap specimen. The model was tested on a macro-scale by reconstructing five fall accidents using subject-specific head models. Linear fracture patterns were predicted in moderate agreement with PMCT scans and forensic analysis for the majority of cases. The model demonstrated high sensitivity to the head mass distribution, the impact velocity and the constitutive variables of cortical bone. The presented procedure to reconstruct occurrences of skull fractures can be used for skull fracture predictions in various applications where linear fracture is dominant.

As continuations of this study, the model should also be investigated in its capability to predict more complex fracture patterns in impacts at higher/lower ranges of velocities, for other modes of fracture (e.g., depressed fractures) and for wider age groups. Most importantly, the model should be evaluated for its pass/fail (fracture/no fracture) predictive ability. In the present set of cases, no fractures were intervening with sutures. However, for future studies, investigating the role of cranial sutures might be of relevance. Adding shear tabulated curves or subject-specific parameters might also be possible study scopes. With further validation, this method could be used for developing skull fracture criteria and potentially help forensic pathologists to infer causes of skull fracture.

## Figures and Tables

**Fig. 10:**
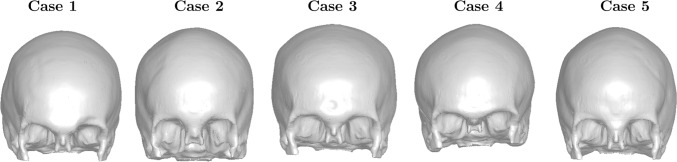
Frontal view of the subject-specific skull models, Case 1 to 5

**Fig. 11:**
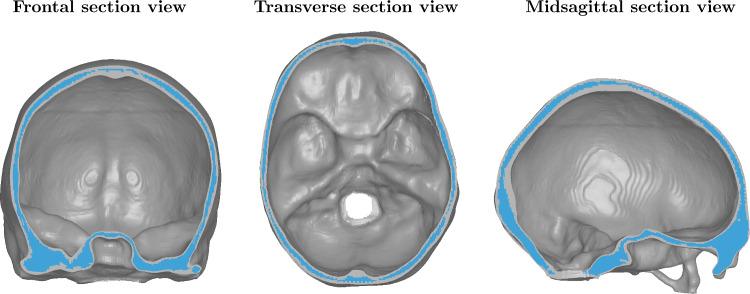
Section views of the skull model (Case 1). The diploë is illustrated in blue

**Fig. 12:**
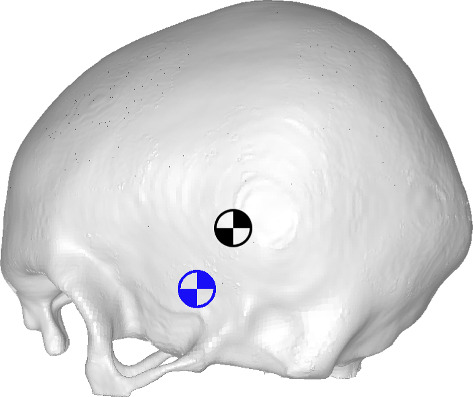
Center of mass shown in a lateral view of the subject-specific head model (in black) and the corresponding CoG reported in literature (in blue). This CoG is illustrated with Case 1 head model

**Fig. 13:**
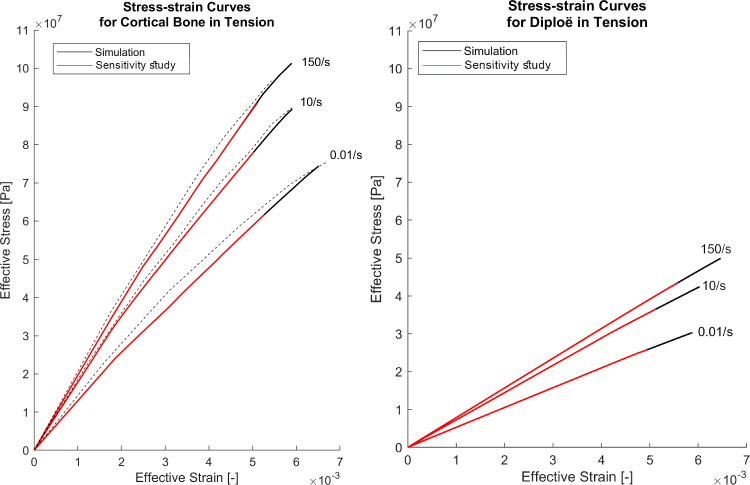
Stress–strain curves for sensitivity study by scaling failure strength

**Fig. 14:**
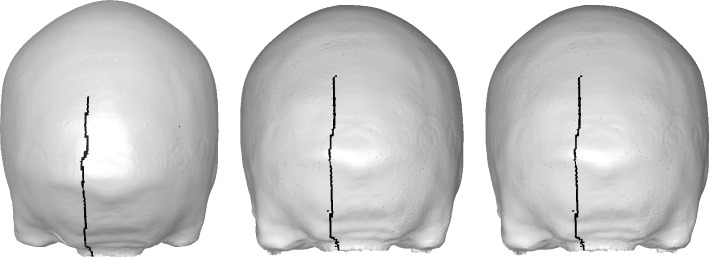
Case 2 simulated with 0.40 Poisson’s ratio of the cortical bone (middle) and with an increased shear modulus of the diploë (right). Reference skull fracture (without increase in Poisson’s ratio or shear modulus) is shown to the left

**Fig. 15:**
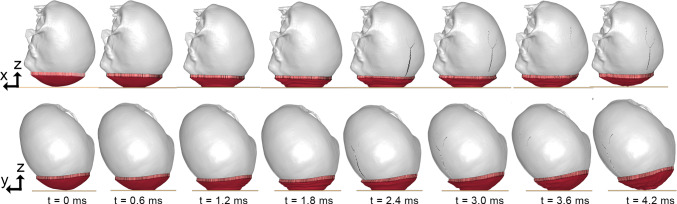
Case 4 simulation time steps

**Fig. 16:**
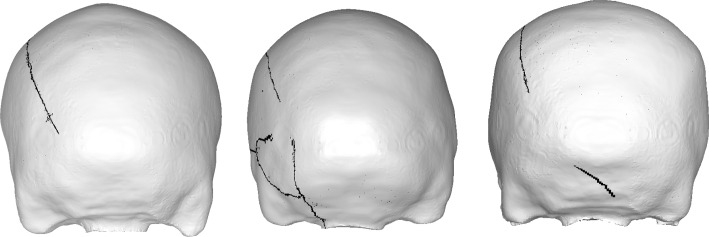
Case 4 simulated with Case 4 (left), Case 1 (middle) and Case 3 (right) subject-specific head models

**Fig. 17:**
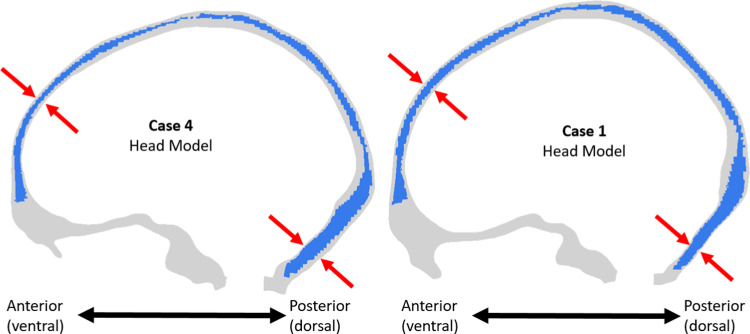
Sagittal cross section of head model of Case 4 and Head model of Case 1. Not the obvious thickness differences in the frontal and occipital bone. Diploë is shown in blue

## Data Availability

The data on cases are not available due to confidentiality. The ADAPT model and morphing algorithm used to conduct the analyses presented in this study are not available for public use due to intellectual property rights, but the models are described in detail in the manuscript, as well in referenced publications.

## Appendix A

### A.1 Subject-specific head models with cross section views

In Table 4, the minimum Jacobians and Dice values for all subject-specific head models are presented. In Fig. 10, the subject-specific head models are depicted in an anterior view. In Fig. 11, cross section views of the cranium of the subject-specific head model for Case 1 are provided. Note the thickness ratios of diploë and cortical bone. In some regions, the diploë is more announced, while it is almost non-existent in others. The division of diploë-cortical bone is originally based on reconstructions of the generic ICBM152 template generated from MRI scans of 152 healthy adult subjects (Fonov et al. 2009). In this study, the inner and outer skull surfaces have been morphed and the diploë-cortical bone thickness ratio has been distorted according to the applied displacement field. The center of gravity (CoG) of the head models was situated relatively close to the reported CoG of human cadaver heads (0.83 cm anterior of the auditory meatus and 3.13 cm superior to the Frankfurt plane) (Yoganandan et al. 2009). The reported CoG coordinates are illustrated together with the measured CoG for Case 3 in Fig. 12.

**Table 4:** Minimum Jacobians and Dice values for the subject-specific head models

	Min. Jacobian	Dice Skull	Dice Skull
	Head Model [-]	Selected* [-]	Total average [-]
Case 1	0.19	0.88	0.81
Case 2	0.18	0.90	0.82
Case 3	0.31	0.89	0.82
Case 4	0.12	0.89	0.81
Case 5	0.14	0.87	0.84

*Here, the average Dice value is presented excluding the foramen magnum and nasal bone of the ADAPT model

### Stress–strain curves for sensitivity study

A sensitivity analysis was performed by simulating Case 2 with modified simulation conditions. One simulation was performed with reduced diploë strength, and one with reduced cortical strength. In Fig. 13, the stress–strain curves for the reduced diploë and cortical bone is presented in relation to the original stress–strain curves.

### Poisson’s ratio and shear modulus test

In Fig. 14 below, Case 2 was simulated with the cortical bone having a Poisson’s ratio of 0.40 instead of 0.20. In a second simulation, Case 2 was computed having an increased shear modulus for the diploë. Here, the shear modulus was the same as of the cortical bone. As suggested by the depictions, no change in the fracture line was observed.

### Case 4: Discussion

Case 4 is a special case in this study. A linear fracture was predicted, but the fracture propagation differs substantially from what we see in PMCT. The FE model suggests that the fall induced a linear skull fracture progressing anteriorly-superiorly from the impact site on the parietal bone. The actual fracture seen in PMCT extended inferiorly from the impact site in the occipital bone toward the foramen magnum. Before trying to explain the simulation-PMCT discrepancy, it should be emphasized, that even though the fracture does not correspond to the real-world fracture, the FE head model does in fact accurately predict the actual occurrence of a linear skull fracture. Case 4 simulation thus gives us valuable information about the possible mode of injury, even though the fracture propagation could be questionable. Since propagation and initiation of skull fracture have not been accounted for in previous fracture prediction attempts, the results of this study are especially noteworthy. On that note, there could be several explanations for the discrepancy between fracture lines seen in simulation and PMCT.

First of all, studying the head impact simulation, the head model rotates toward its left side after the impact (see Fig. 15). Comparing the head movement with the full-scale HBM simulation, the post-impact head rotation about x-axis is not represented. It is hence possible, that the simplifications made in the subject-specific head model affects the post-impact head movement. Such simplifications are, e.g., the missing face and neck, or the manipulated head mass distribution. The center of gravity is not situated as low dorsally as suggested by literature (Yoganandan et al. 2009) (see Appendix), affecting the overall inertia of the head. The post-impact kinematics can also be affected by other assumed variables: friction coefficients, the impacting ground material properties, the thickness of the scalp. It can be hypothesized, that the post-impact kinematics determines where-to the fracture propagates.

A second possible explanation to the Case 4 fracture progressing anteriorly-superiorly from the impact site on the parietal region might be found in the head model. For comparison, Case 4 impact was simulated using the subject-specific head models of Case 1 and 3. The resulting fracture patterns are shown in Fig. 16. An initial fracture line progressing anteriorly-superiorly from the impact site on the parietal bone was present in all instances. A secondary fracture line on the occipital bone of the head was induced in Case 4 simulated with Case 1 and 3 head models. This suggests that a fracture line propagating toward the parietal bone is not unlikely with the Case 4 set of applied boundary conditions. Another interesting takeaway from Fig. 16 is how much the head geometry influences the fracture line. In the three simulations, the only differing factor is the skull geometry and scalp thickness. The comparison emphasizes the importance of accounting for subject-specific morphology. The morphology might also explain why we do not see any secondary fracture line propagating toward the foramen magnum in the original simulation of Case 4. Looking at the thickness of the Case 4 head model in the parietal bone, we see that it is evidently thinner compared to Case 1 and 3 head model, and the cranial bone in the occipital region is thicker. Sagittal cross sections of Case 4 and Case 1 head model are seen in Fig. 17. One logical explanation to the simulated fracture pattern is that the fracture is more inclined to propagate toward the thinner, i.e., weaker, parts of the skull.

Lastly, an important aspect to keep in mind is that the *exact* time-history of the victim’s fall is not known. The victim most likely fell from standing, and since no relevant injuries were documented, the free fall was most likely “clean”, without any other body parts intervening in the fall. The HBM reconstruction is a simplification of reality. In the real-world accident, the head impact in Case 4 might have been affected by factors not incorporated into the HBM reconstruction: in this particular case, the victim might have fallen from a stance different from the HBM fall reconstruction, he might have activated certain muscles (e.g., in the neck) that affected the impact kinematics, or the victim might have induced rotations to the head during fall. This would not have been captured in the HBM reconstructions. The mentioned slight differences in head impact kinematics might affect the path of propagation of the linear fracture that we see in the simulation, and the assumptions made could have affected the fracture more or less in this particular case.
